# Draft Genome Sequence of Aquitalea sp. Strain MWU14-2217, Isolated from a Wild Cranberry Bog in Provincetown, Massachusetts

**DOI:** 10.1128/MRA.01493-18

**Published:** 2018-11-29

**Authors:** Ghazal Ebadzadsahrai, Scott Soby

**Affiliations:** a Biomedical Sciences Program, College of Graduate Studies, Midwestern University, Glendale, Arizona, USA; b Biomedical Sciences Program, College of Graduate Studies and College of Veterinary Medicine, Midwestern University, Glendale, Arizona, USA; Georgia Institute of Technology

## Abstract

Aquitalea sp. strain MWU14-2217 was isolated from wild cranberry bog soils in the Cape Cod National Seashore. The draft genome is 4.3 Mbp with 4,133 coding sequences and contains predicted genes for phenazines, colicins, siderophores, and putative exporters of these compounds and genes responsible for motility and biofilm formation.

## Abstract (precis)

Aquitalea sp. strain MWU14-2217 was isolated from wild cranberry bog soils in the Cape Cod National Seashore.

## ANNOUNCEMENT

The genus Aquitalea was described in 2006 as a member of the Neisseriales, now placed in the family Chromobacteriaceae, and is closely related to the genus Chromobacterium (1). This genus includes three recognized species, all of which were isolated from aquatic environments (1–4). Despite the importance of wetland bacteria in geochemical processes, little is known about their taxonomy or environmental functions. As part of a culture-dependent survey of bacteria from wetland bogs, MWU14-2217 was isolated along with a number of previously uncharacterized Pseudomonas spp. and Chromobacterium spp. from wild cranberry bogs in the Cape Cod National Seashore in Massachusetts. The discovery of new species within well-established genera suggests an important if underappreciated example of microbial evolution in these critical ecosystems.

**FIG 1:**
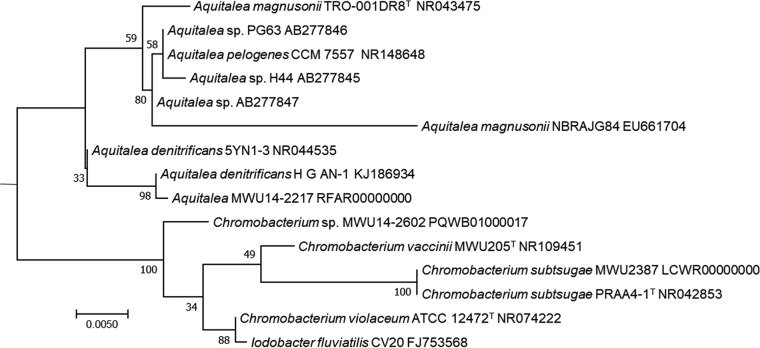
16S rRNA phylogeny of *Aquitalea* sp. strain MWU14-2217. The evolutionary history of this *Aquitalea* sp. strain was inferred in MEGA7 (13) by maximum likelihood based on the Kimura 2-parameter model (14) with a discrete gamma distribution (5 categories; +*G* parameter, 0.7410), a complete deletion of gaps and missing data, and a rate variation model that allowed for some evolutionarily invariable sites (+*I*, 82.42% of sites). There were a total of 969 positions in the final data set. Initial trees for the heuristic search were obtained from Neighbor-Join and BioNJ algorithms applied to a matrix of pairwise distances estimated using maximum composite likelihood. The tree with the highest log likelihood (−1,883.98) was drawn to scale with branch lengths measured in the number of substitutions per site. Bootstrap values from 500 samplings are indicated next to branches. *Aquitalea* sp. sequences were retrieved from GenBank by taking the bacterial 16S rRNA RefSeq targeted locus project sequence for each species. *Chromobacterium* and *Iodobacter* were included as outgroups. MWU 14-2217 clusters with the *A. denitrificans* subgroup, but in the absence of a genomic sequence for *A. denitrificans*, the exact taxonomic placement of MWU 14-2217 cannot be determined.

Soil samples were plated onto King’s medium B (KMB) supplemented with ampicillin and cycloheximide, and then single colonies were purified three times on KMB. MWU14-2217 was provisionally placed in the genus Aquitalea by 16S rRNA gene similarity (∼99%) with other members of the genus by phylogenetic analysis (Fig. 1). For genome sequencing, MWU14-2217 was grown in KMB broth overnight for genomic DNA (gDNA) isolation (DNeasy blood and tissue kit, Qiagen). gDNA was sheared to ∼600 bp to generate libraries on an Apollo 384 liquid handler (Wafergen, Kapa Biosystems KK8201). Resulting DNA fragments were end repaired, A tailed, ligated to indexes/adapters (catalog number 520999; Bioo), and cleaned using AMPure beads (Agencourt Bioscience/Beckman Coulter). Samples were amplified with Kapa HiFi enzyme, and the resultant libraries were assessed by quantitative PCR (Kapa library quantification kit KK4835), pooled, and sequenced on the Illumina MiSeq platform in 2 × 300-bp and 2 × 150-bp paired-end flow cells. All read file data sets were combined, trimmed, partially assembled, and annotated using the Comprehensive Genome Analysis feature of the PATRIC (version 3.5.26) website (https://patricbrc.org/) with default parameters (5). The sequence consisted of 4,335,744 bp (G+C content, 60.26%) within 25 contigs. The largest contig is 755,022 bp, and the *N*_50_ value is 448,300 bp, with a sequence coverage of 151×. The orthologous average nucleotide identity (ANI) was less than 86% (6, 7), and the digital DNA-DNA hybridization (dDDH) (8, 9) was <30% with Aquitalea magnusonii (GenBank accession number AP018823) (2) and Aquitalea pelogenes (GenBank accession number LNQV00000000) (4), the only two genomic sequences available for this genus. The closest relative based on the 16S rRNA sequence is Aquitalea denitrificans (Fig. 1), but at this time, no whole-genome sequence is available.

Gene number and functional predictions were made using the RASTtk function of PATRIC with default settings (6). MWU14-2217 contained 4,133 predicted protein-coding genes (4,680 by NCBI annotation), with 76 tRNA and 9 rRNA operons, 23 chemotaxis (*che*) and 5 aerotaxis genes, the multidrug efflux pump genes *mdtABC* (10) and *emrAB* (11), the macrolide-specific *macAB* (12) efflux pump, and an exoprotease exporter gene. Although the bacterium is not fluorescent, a phenazine-like biosynthesis gene (*phzF*) is present, as well as a colicin V production protein and a homoserine lactone efflux protein.

### Data availability.

This whole-genome shotgun project has been deposited at DDBJ/EMBL/GenBank under the accession number RFAR00000000 and in the SRA database under accession number SRP167089.
